# Sarcopenia and Frailty in Liver Cirrhosis

**DOI:** 10.3390/life11050399

**Published:** 2021-04-27

**Authors:** Hiroki Nishikawa, Shinya Fukunishi, Akira Asai, Shuhei Nishiguchi, Kazuhide Higuchi

**Affiliations:** 1 The Second Department of Internal Medicine, Osaka Medical and Pharmaceutical University, Takatsuki, Osaka 569-8686, Japan; in2104@osaka-med.ac.jp (S.F.); in2108@osaka-med.ac.jp (A.A.); higuchi@osaka-med.ac.jp (K.H.); 2 Department of Internal Medicine, Division of Gastroenterology and Hepatology, Hyogo College of Medicine, Nishinomiya, Hyogo 663-8501, Japan; 3 Kano General Hospital, Osaka 531-0041, Japan; nishiguchi@heartfull.or.jp

**Keywords:** sarcopenia, frailty, liver cirrhosis, pathophysiology, guidelines

## Abstract

Skeletal muscle is the largest organ in the body, and skeletal muscle atrophy results from a shift in the balance of protein synthesis and degradation toward protein breakdown. Primary sarcopenia is defined as a loss of skeletal muscle mass and strength or physical function due to aging, and secondary sarcopenia is defined as a loss of skeletal muscle mass and strength or physical function due to underlying diseases. Liver cirrhosis (LC) is one of the representative diseases which can be complicated with secondary sarcopenia. Muscle mass loss becomes more pronounced with worsening liver reserve in LC patients. While frailty encompasses a state of increased vulnerability to environmental factors, there is also the reversibility of returning to a healthy state with appropriate intervention. Several assessment criteria for sarcopenia and frailty were proposed in recent years. In 2016, the Japan Society of Hepatology created assessment criteria for sarcopenia in liver disease. In Japan, health checkups for frailty in the elderly aged 75 years or more started in April 2020. Both sarcopenia and frailty can be adverse predictors for cirrhotic patients. In this review article, we will summarize the current knowledge of sarcopenia and frailty in LC patients.

## 1. Liver Cirrhosis and Sarcopenia: Pathophysiology and Prognosis

Skeletal muscle is the largest organ in the body, and it accounts for about 50% of the body’s total protein mass and maintains energy expenditure throughout the body [1]. It is necessary to consider skeletal muscle as an endocrine organ [2]. Skeletal muscle mass is maintained by a delicate balance between muscle protein catabolism and muscle protein anabolism. Skeletal muscle atrophy results from a shift in the balance of protein synthesis and degradation toward protein breakdown [3]. Sarcopenia refers to a loss of skeletal muscle mass, muscle strength or physical function [4]. When sarcopenia was first proposed by Rosenberg in 1989, it referred only to the loss of muscle mass [5], but later, the quality of muscle mass was also emphasized, leading to the current definition. The year 1989 was also the year of the discovery of the hepatitis C virus, and, in a sense, it can be said to be a period of change. In recent years, a large amount of evidence on sarcopenia was accumulated, and the disease was registered in ICD-10, and sarcopenia is now recognized as a disease rather than a clinical entity [6]. Primary sarcopenia is defined as a loss of skeletal muscle mass and strength or physical function due to aging, and secondary sarcopenia is defined as a loss of skeletal muscle mass and strength or physical function due to underlying diseases [7]. As the disease itself forces rest, sarcopenia due to disease-related physical inactivity may also occur. In a large study of 4811 elderly Japanese subjects, the prevalence of sarcopenia was shown to be 7.5% [8]. The complication rate of sarcopenia in patients with liver cirrhosis (LC) was reported to be 30–70%, which is clearly higher considering that the complication rate of sarcopenia in inflammatory bowel diseases, typical diseases causing secondary sarcopenia, is about 20% [9,10]. In LC patients, the annualized skeletal muscle loss rate was 1.3% in Child-Pugh A, 3.5% in Child-Pugh Band 6.1% in Child-Pugh C [11]. Muscle mass loss became more pronounced with worsening liver reserve in cirrhotic patients, which is higher than the 1% annualized loss of muscle mass in the average elderly person [11,12].

The pre-sarcopenia stage, in which only muscle strength is reduced, is sometimes referred as dynapenia, and the importance of muscle quality is also receiving increasing attention [13,14,15]. In a retrospective study of 411 patients with chronic liver disease (CLD) at our institution, the incidence of liver disease-related events (ascites, encephalopathy, varices, liver failure, hepatocellular carcinoma (HCC), etc.) was significantly higher in patients with reduced grip strength (GS) than in patients without reduced GS, and reduced GS was an independent risk factor for the liver disease-related events [13]. On the other hand, the prognostic impact of reduced muscle mass cases was not as great as that of GS compared to non-reduced muscle mass cases [13]. In another study of our 389 CLD patients using the short form 36 health survey questionnaire (SF36), the physical component summary score was significantly lower in patients with reduced GS than in patients with non-reduced GS, while the same result was not observed in patients with reduced muscle mass. There was no significant difference in the mental component summary score between reduced GS vs. non-reduced GS cases, and reduced muscle mass vs. non-reduced muscle mass cases [14]. These results suggest that muscle strength is more closely related to physical function than muscle mass, and that there is no difference in mental function. On the other hand, sarcopenia with increased fat mass (sarcopenic obesity, for which there is no established standard value at present) has attracted attention as a poor prognostic factor in LC patients [16]. The concept of metabolic syndrome-related sarcopenia was also reported, and attention should be paid not only to muscle mass and strength, but also to fat mass, waist circumference, hypertension, hyperlipidemia and diabetes mellitus [17].

The mechanism of sarcopenia in LC patients was reported to involve protein-energy malnutrition (PEM, as defined by hypoalbuminemia and low non-protein respiratory quotient, as assessed by indirect calorimetry), which is characteristic of cirrhotic patients, signaling related to protein synthesis and degradation, dysbiosis and myokines such as myostatin (cytokine with the inhibition of muscle protein synthesis), the ubiquitin-proteasome pathway, insulin resistance and the sex hormone (testosterone, etc.) [18,19,20,21]. In particular, muscle hypertrophy in myostatin knockout mice is surprisingly marked, indicating how myostatin is involved in the regulation of muscle protein growth [22]. Our data showed that serum myostatin level increased significantly with worsening liver reserve (Child-Pugh A vs. Child-Pugh B or C, *p* = 0.0011), suggesting that more potent inhibition of muscle protein synthesis occurs with worsening liver reserve [19]. Myostatin secretion in muscle is also increased by elevated serum ammonia [23]. In our data, there was a significant positive correlation between serum myostatin level and serum ammonia level in LC patients (correlation coefficient: *r* = 0.5856 (*p* < 0.0001) in male, *r* = 0.3922 (*p* < 0.0001) in female) [19]. Therefore, myostatin is thought to be regulated by serum ammonia. In addition, decreased diversity of intestinal bacteria associated with dysbiosis, increased intestinal permeability, decreased short-chain fatty acids as a source of energy, and decreased antioxidant activity, can exacerbate the pathogenesis of cirrhosis [21]. Branched chain amino acids (BCAAs) are among the most anabolic of the essential amino acids, and the role of BCAAs (especially leucine) in protein synthesis in skeletal muscle is significant [24,25]. On the other hand, sarcopenia can be linked to osteoporosis [26]. Although both sarcopenia and osteoporosis affect different organs and tissues, they are closely interrelated through common factors, such as genetic factors, endocrine hormones, nutritional status and daily activities [27,28]. LC patients with both osteoporosis and sarcopenia (i.e., osteosarcopenia) have elevated risk of vertebral fractures [26,29].

In most of the studies on the relationship between sarcopenia and prognosis in liver disease reported from Japan, the prognosis of sarcopenic patients was significantly worse than that of non-sarcopenic patients, and sarcopenia is considered to be a poor prognostic factor in liver disease [30]. LC-related complications such as ascites, encephalopathy and varices can be linked to sarcopenia progression [13,31]. Sarcopenia should be considered as an important prognostic factor in LC patients as well as Child-Pugh classification and albumin-bilirubin (ALBI) grade, and the evaluation of sarcopenia has become an essential part of daily practice for CLD patients [30,32]. In recent years, the term “gut-liver-muscle axis”, which refers to the organ-organ relationship between the gut, liver and skeletal muscle, was frequently used [33].

## 2. JSH Guidelines for Sarcopenia in Liver Disease and Japan Evidence-Based Clinical Practice Guidelines for Liver Cirrhosis 2020 (3rd Edition)

In 2016, the Japanese Society of Hepatology (JSH) proposed sarcopenia criteria specific to liver disease [30]. Prior to this, it was recognized that sarcopenia is a poor prognostic factor in liver disease, but the definition of sarcopenia itself and the reference values for muscle mass and other parameters used for the assessment of sarcopenia varied even among reports in Japan, causing some confusion in a sense. Therefore, the JSH formed a working group on sarcopenia assessment criteria specific to liver disease with the intention of standardizing the criteria in Japan [30]. Compared to the sarcopenia assessment criteria used in other countries such as Asia and Europe, the JSH criteria has the following features: (1) elimination of age limit (because secondary sarcopenia may occur due to the pathology of liver disease unrelated to age), (2) elimination of walking speed (WS) because of the complexity of the measurement of WS in daily clinical practice and (3) specification of reference values for muscle mass on computed tomography (CT) because CT is frequently used in CLD patients [30]. The JSH criteria for sarcopenia was a novel and simple system that took into account the characteristics of daily clinical settings in liver disease.

Screening procedure of the JSH is quite simple: First, GS is screened, and muscle mass is measured by bioelectrical impedance analysis (BIA) or CT for cases below the reference values of GS (26 kg for male and 18 kg for female). Cases below the reference values of BIA or CT are then diagnosed as sarcopenia [30]. In our study of 636 CLD patients in whom muscle mass was measured using the BIA method, skeletal muscle indexes (SMIs) of 242 patients (38.1%) were below the reference values (7.0 kg/m^2^ for male and 5.7 kg/m^2^ for female in both the first edition of the Asian Working group for Sarcopenia (AWGS) criteria and the JSH criteria) [30,34], and the proportion of patients with low muscle mass increased with increasing age. Additionally, SMIs of about 25% of patients younger than 65 years were below the reference values [30]. With regard to WS, only 17 (4.8%) of our 356 CLD patients had a WS below the initial AWGS criterion (0.8 m/s, the speed at which a person can manage to cross a crosswalk safely) [34], indicating that WS may not be suitable as an initial screening for sarcopenia [35]. These results do not deny the usefulness of measuring WS as an indicator of physical function. In addition, the risk of falling should be taken into consideration when conducting the walking test. On the other hand, 51 of our 356 CLD patients (14.3%) met the criterion for WS in the revised AWGS (1.0 m/s) [36]. The fact that the reference value for WS was raised to 1.0 m/s in the revised AWGS seems to be a reasonable decision in light of our data [36]. As mentioned earlier, the significance of GS has been attracting attention [15], and this may indicate the validity of first screening by GS when determining sarcopenia.

Since the proposal of assessment criteria for sarcopenia in liver disease by the JSH, many discussions were done based on the JSH criteria in Japan, and the fact that many overseas researchers cited the article on the JSH criteria shows that the JSH criteria for sarcopenia in patients with liver disease are becoming widely recognized (https://onlinelibrary.wiley.com/doi/full/10.1111/hepr.12774, 167 citations in 3 April 2021). A working group is currently studying the revision of the JSH criteria. On the other hand, the term “sarcopenia” is not found at all in the Japan Evidence-based Clinical Practice Guidelines for Liver Cirrhosis 2015 (2nd edition) [37]. One of the main features of the Japan Evidence-based Clinical Practice Guidelines for Liver Cirrhosis 2020 (3rd edition) is the new introduction of the term “sarcopenia”. In this guideline, the assessment of sarcopenia is mandatory (level A) in LC patients. It also includes the determination of sarcopenia in the flow chart for nutritional therapy, and provides a nutritional guidance policy for LC patients with or without (1) a serum albumin level of 3.5 g/dL or less, (2) a Child-Pugh classification of B or C or (3) sarcopenia.

## 3. Sarcopenia Assessment Criteria: Revision of Assessment Criteria in Other Countries

The AWGS criteria for sarcopenia and the European Working Group for Sarcopenia in Older People (EWGSOP) criteria for sarcopenia underwent considerable changes in the revised version compared to the original version [36,38]. The JSH criteria was created based on the concept of the original version of the AWGS criteria for sarcopenia [30,34]. First of all, the revised AWGS criteria take into account the clinic level, where muscle mass cannot be measured, and sarcopenia can be determined by only muscle strength and physical function (e.g., WS). In addition, the reference value of GS for men was revised from 26 kg in the first edition to 28 kg, and the reference value of WS was revised to 1.0 m/s, as mentioned above [34,36]. This seems to have made it possible to pick up a wide range of suspected cases of sarcopenia and intervene to improve them. As an initial screening for sarcopenia, the revised AWGS criteria recommends measurement of calf circumference (CC, reference values: 34 cm for men and 33 cm for women) and SARC-F (a method of diagnosing suspected sarcopenia when the total score of strength (S), assistance walking (A), rising from a chair (R), climbing stairs (C) and falling (F) is 4 points or more, with 0, 1 and 2 points, respectively) [36]. CC and SARC-F can be useful initial screening tools for sarcopenia in CLD patients [39,40,41]. However, it should be noted that SARC-F was reported to be less sensitive (about 0.4–0.5) [42]. It also recommends assessment of the severity of sarcopenia (defined as severe sarcopenia when muscle strength, muscle mass and physical function are all decreased) [36]. It is noteworthy that the revised EWGSOP also recommends initial screening for sarcopenia using SARC-F and further recommends muscle mass assessment by CT or magnetic resonance imaging (MRI) as in the current JSH criteria, as well as the severity assessment of sarcopenia as in the revised AWGS [38]. Table 1 shows the reference values for the revised EWGSOP, the JSH and the revised AWGS sarcopenia assessment criteria. The first edition of the JSH sarcopenia criteria was published in 2016, and a revised edition has not yet been published. However, it is hoped that a refined revised edition will be published in line with the revised EWGSOP and AWGS guidelines.

The finger-circle test is also useful for assessing muscle mass [43]. The finger-circle test is a method in which a circle is formed between the thumb and index fingers of both hands, and the calf is evaluated in three stages: “cannot be enclosed (Bigger)”, “just enclosed (Just-fits)” and “gap (Smaller)”. It is a very simple evaluation method that can be performed anywhere, and its usefulness in liver diseases was reported in Japan [43]. In our data of 202 CLD patients, the percentage of sarcopenia was 3.5% in Bigger, 18.2% in Just-fits and 33.3% in Smaller patients, suggesting the usefulness of the finger-circle test [44]. The finger-circle test is particularly useful at the clinic level, where CT or BIA cannot be used. The Japanese Association on Sarcopenia and Frailty also recommends screening for sarcopenia with the finger-circle test (http://jssf.umin.jp/jssf_guideline2017.html, accessed on 1 April 2021, written in Japanese) [45]. It is important to pick up suspected cases of sarcopenia as a first step.

## 4. Intervention for LC Patients with Sarcopenia

Interventions for sarcopenia complicated by cirrhosis include: 1. nutritional intervention, 2. exercise intervention, 3. nutritional and exercise intervention and 4. drug therapy, etc. [46]. An observational study of 568 elderly Japanese subjects showed a significant negative correlation between the number of steps and activity using accelerometers continuously over five years and the development of sarcopenia [47]. Based on these findings, it is quite natural to assume that exercise is the first step in improving sarcopenia, even in cirrhosis. However, although the Japan Evidence-based Clinical Practice Guidelines for Liver Cirrhosis 2020 (3rd edition) suggests exercise therapy and nutritional therapy such as BCAA preparations, the evidence level of them is only level C based on the results of past studies, and there is still room for further study. In particular, at present, exercise intervention for patients with Child-Pugh C is not recommended due to lack of evidence, although a recent meta-analysis including eleven studies (five randomized controlled trials, five observational studies, one case-control study) reported that exercise intervention improved VO_2_ peak, anaerobic threshold, 6-min walk distance, muscle mass, muscle function and QOL in compensated or decompensated LC patients [48]. Exercise intervention in elderly LC patients or LC patients with varices should be considered after thorough evaluation of the condition and consideration of safety, taking into account the possibility of falls or varices rupture. On the other hand, earlier intervention of exercise in LC patients with pre-sarcopenia may be beneficial because in the real-world clinical settings, continuation of exercise is often difficult in LC patients with reduced sarcopenia.

Although pharmacotherapy is not recommended or suggested in the Japan Evidence-based Clinical Practice Guidelines for Liver Cirrhosis 2020 (3rd edition), there are promising drugs such as l-carnitine or rifaximin (antibiotics) with ammonia-improving effects, and the results of future studies on the effect of these drugs on sarcopenia are awaited [21]. L-carnitine plays an important role in burning fatty acids and converting them into energy such as ATP, and when carnitine is deficient, muscles do not produce enough energy, resulting in muscle weakness, muscle pain and muscle cramps [49]. In animal studies, sarcopenia was shown to improve via ammonia clearance amelioration, and the same was reported in clinical studies on l-carnitine [23,50,51]. It is also known that cirrhosis is associated with a higher prevalence of dysbiosis, which leads to hyperammonemia due to alterations in the gut microbiota [52]. Hyperammonemia increases the secretion of myostatin, a muscle growth inhibitor, and causes the development of sarcopenia [19]. Higher serum myostatin level in LC patients can be a poor prognostic factor [19]. In LC patients, rifaximin improves dysbiosis and is expected to improve sarcopenia [53,54]. On the other hand, it should be noted that serum zinc (Zn) levels tend to decrease in LC patients with sarcopenia [55,56]. Zn deficiency in LC often accompanies various functional disorders, and enhances intestinal inflammation through macrophage activation [57]. Decreased Zn level in LC can be associated with decreased ammonia clearance [58]. In our previous study, the frequency of sarcopenia was significantly higher (27.2%) in the low Zn group [55]. In another study on Zn, we found that the prognosis of LC patients was well stratified when we classified baseline serum Zn levels into three groups: below 60 μg/dL as Zn deficient, between 60 μg/dL and 80 μg/dL as potentially Zn deficient and between 80 μg/dL and 130 μg/dL as normal (serum Zn normal group had the best prognosis, and Zn deficient group had the worst prognosis) [58]. Whether maintaining normal serum Zn levels in LC patients with hypozincemia and sarcopenia improves prognosis through the improvement of sarcopenia requires further investigation. Recently, the usefulness of cancer rehabilitation in HCC patients was reported from Japan [59]. This study was a prospective observational study of 152 HCC patients who underwent transcatheter arterial chemoembolization (85 HCC patients in the cancer rehabilitation group vs. 67 HCC patients in the control group), and there was a significant difference in the overall survival between the two groups even after propensity score matching. Another recent study reported that exercise therapy improves frailty in HCC patients [60]. Myostatin-specific antibody can be promising in LC patients. It can improve muscle strength [61]. It was reported that vitamin D receptors are expressed in skeletal muscle cells, suggesting that vitamin D may have a direct effect on skeletal muscle [62]. LC patients often have low vitamin D levels, and vitamin D supplementation in LC patients is also promising for improving sarcopenia [63]. Testosterone therapy in male LC patients may be beneficial for the improvement of sarcopenia [64]. 

## 5. Frailty in Liver Disease: Its Definition and Prevalence

Japan is an aging society. In the elderly, physiological reserve gradually decreases, and homeostasis is lost. Frailty is a syndrome commonly observed in the elderly [65]. It encompasses a state of increased vulnerability to environmental factors, but with the reversibility of returning to a healthy state with appropriate intervention [66,67,68]. There are physical, psychological and social factors that contribute to frailty [65]. Many older persons transition from a state of well-being through frailty to a state of needing care [65]. In addition to a higher risk of developing nursing care needs, elderly people with frailty have a poorer life expectancy and a higher risk of hospitalization, falls and fractures compared to healthy people [66,69]. Polypharmacy, which is often a problem in the elderly, can be a risk factor for developing frailty [70]. In Japan, health checkups for frailty in the elderly aged 75 years or more started in April 2020, and the start of health checkups for frailty is a major turning point, because health checkups up to fiscal 2019 focused on measures against metabolic syndrome. The questionnaire used in the health checkups for frailty consists of health status, mental health status, eating habits, oral function, weight change, exercise and falls, cognitive function, smoking, social participation and social support to assess the health status of the elderly comprehensively (http://kaigo.homes.co.jp/manual/healthcare/kaigoyobo/flailexamination, accessed on 1 April 2021, written in Japanese). As a certain number of Japanese CLD patients are expected to be included in the target population of health checkups, frailty in liver diseases is also an important issue as well as sarcopenia [71,72]. Its clinical significance was increasingly recognized in CLD patients in recent years; however, frailty is not routinely measured in CLD patients [66].

Fried et al. defined frailty as meeting three or more of the following criteria: Shrinking, Weakness, Exhaustion, Slowness and Low activity, and pre-frail as meeting one or two of these criteria [65]. The prevalence of frailty among community-dwelling elderly persons was 5.6% in Japan [73]. In our study of CLD patients using Fried criteria (*n* = 341, 122 LC cases, median age = 66 years), 46 (13.5%) were frailty and 187 (54.8%) were pre-frail, with the frequency of frailty increasing with age (*p* = 0.0002), and the proportion of LC patients in frailty patients was higher than that in non-frailty patients (67.4% (31/46) vs. 30.9% (91/295), *p* < 0.0001) [74]. These results mean that frailty, like sarcopenia, has aspects that are caused by the disease condition. The term “secondary frailty” does not exist, but it may be used in the future. It should also be noted that there are many pre-frail CLD patients. Pre-frail is a reversible condition similarly to frailty, and the significance of early intervention in the pre-frail stage needs to be examined [75]. On the other hand, the frequency of frailty was not affected by body mass index (BMI) in our data [74]. Similar results were reported by Lai, et al. [76]. In our study, we found that the frequency of sarcopenia increased with decreasing BMI; in other words, lower BMI can be a risk factor for sarcopenia, and the discrepancy between the two is interesting (Table 2) [74]. Although sarcopenia is the main component of physical frailty, the differences between the two should also be recognized. Sarcopenia is only an assessment based on muscle quality and quantity [77]. Frailty, on the other hand, includes factors other than muscle quality and quantity, such as fatigue and psychological factors [78]. This difference may be one reason for the discrepancy between sarcopenia and frailty in CLD patients. Frailty is a complex clinical entity characterized by functional decline and reduced physiological reserve [78]. We should not forget that liver disease is a chronic disease, and that the negative factors of “psychological and social burdens associated with prolonged illness” are associated with frailty.

The Liver Frailty Index (LFI) is a diagnostic method of frailty specific to liver diseases [79]. The LFI is composed of three performance-based tests (GS, chair stands and balance) [79]. Its usefulness in liver transplant patients was reported from overseas, and its usefulness in HCC patients was also reported from Japan [79,80,81,82,83,84,85]. The LFI can be closely linked to muscle atrophy in CLD patients [83]. Wang, et al. reported that in 166 LC patients, 23 (13.9%) had frailty, while in 91 CLD patients without LC, 5 (5.5%) had frailty as assessed by the LFI [80]. In patients undergoing liver transplantation (LT), pre-transplant LFI can be associated with post-transplant robustness [82]. The LFI can be helpful for waitlist mortality in patients awaiting LT [76]. Furthermore, incorporating LFI with MELD-Na can more accurately predict waitlist mortality in patients awaiting LT [86]. Assessment methods for frailty, prevalence of frailty and outcomes in CLD patients reported in recent years are summarized in Table 3.

## 6. Frailty Cycle in LC Patients and Interventions for LC Patients with Frailty

**Figure 1:**
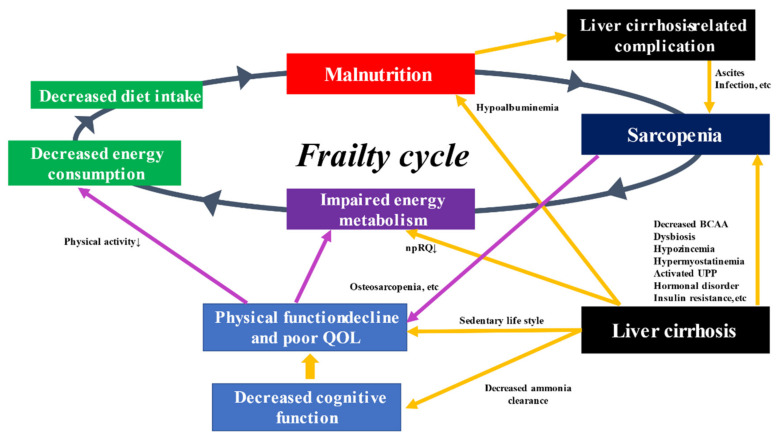
The frailty cycle in patients with liver cirrhosis. BCAA, branched-chain amino acid; npRQ, non-protein respiratory quotient; UPP, ubiquitin-proteasome pathway.

In general, when muscle strength and muscle mass decrease due to aging and other factors, the amount of activity decreases, and energy consumption declines [77]. In addition, a decrease in dietary intake leads to a state of malnutrition due to insufficient intake of protein and other nutrients. Persistent malnutrition leads to weight loss and loss of muscle strength and muscle mass. This vicious cycle (frailty cycle) increases the possibility of falls, fractures or worsening of chronic diseases that may lead to the need for nursing care [87]. The frailty cycle in LC patients is demonstrated in Figure 1.

Most studies on exercise in LC patients predominantly include Child-Pugh A cirrhotic patients, with few data on Child-Pugh B or C cirrhotic patients [88,89,90,91]. Exercise interventions in LC patients were demonstrated to improve exercise capacity, muscle mass and muscle function, as well as QOL and portal hypertension [88,89,90,91]. A recent multi-center study with regard to the in-hospital exercise on frailty in HCC patients (*n* = 181, 114 HCC patients in the exercise group and 67 HCC patients in the control group) reported that in-hospital exercise was an independent factor for the improvement of LFI (odds ratio = 2.38, *p* = 0.0091) [60]. However, the type of exercise that is most beneficial for the improvement of frailty in LC or HCC patients was not established. Furthermore, the outcomes of long-term exercise in LC or HCC patients with frailty were not clarified. Especially in elderly LC patients with frailty, the risk of falls due to exercise can increase, and exercise in LC patients with frailty and varices can lead to the elevated risk of varices rupture [92]. Reports showing an improvement of frailty by nutritional supplementation or pharmacological therapies in LC patients are also currently lacking. 

## 7. Closing Remarks

More than 30 years have passed since the concept of sarcopenia was proposed by Rosenberg in 1989, and criteria for sarcopenia in elderly people were first proposed in Europe in 2010 and in Asia in 2014 [5,7,34]. In Japan, sarcopenia assessment criteria specific to liver disease were first proposed by the JSH in 2016, and based on these criteria, many discussions were conducted, and many findings were obtained [30]. Now, sarcopenia has been newly introduced in the Japan Evidence-based Clinical Practice Guidelines for Liver Cirrhosis 2020 (3rd edition). The progress of sarcopenia research in Japan over the past few years has been remarkable. Considering that sarcopenia is one of the biggest concerns worldwide, and that most people will suffer from sarcopenia at some point in their lives, except for sudden death, the authors believe that academic interest in sarcopenia will never fade. Frailty is a major health problem, and research interest of frailty in LC patients was increasing in recent years as well as in sarcopenia. It is the authors’ sincere hope that the unknown issues related to sarcopenia and frailty will be resolved, and that novel evidence will be created.

## Figures and Tables

**Table 1:** Reference values for the EWGSOP, the JSH and the revised AWGS.

Parameter	Measurement	Revised EWGSOP	JSH	Revised AWGS
Muscle mass	DXA	M: 7.0 kg/m^2^		M: 7.0 kg/m^2^
		F: 5.5 kg/m^2^		F: 5.4 kg/m^2^
	BIA		M: 7.0 kg/m^2^	M: 7.0 kg/m^2^
			F: 5.7 kg/m^2^	F: 5.7 kg/m^2^
	CT (L3 level)		M: 42 cm^2^/m^2^	
			F: 38 cm^2^/m^2^	
Muscle strength or function	Grip strength	M: 27 kg	M: 26 kg	M: 28 kg
		F: 16 kg	F: 18 kg	F: 18 kg
	Walking speed	6 min (400 m)		1.0 m/s
	Chair stand (5 rises)	15 s		12 s
	SPPB	8 point		10 point

EWGSOP, European working group for sarcopenia in older people; JSH, Japanese society of hepatology; AWGS, Asian working group for sarcopenia; M, male; F, female; DXA, Dual-energy X-ray absorptiometry; BIA, bioelectrical impedance analysis; CT, computed tomography; SPPB, Short Physical Performance Battery (Side-by-side-stand, Semi-Tandem Stand and Tandem Stand).

**Table 2:** Comparison of sarcopenia and frailty in CLD patients.

	Sarcopenia	Frailty
Age	tend to be higher age	tend to be higher age
LC status	tend to be affected by LC status	tend to be affected by LC status
Grip strength	tend to be decreased	tend to be decreased
Muscle mass	tend to be decreased	Not always affected by muscle mass
BMI	tend to be decreased	Not always affected by BMI

LC; liver cirrhosis, BMI; body mass index.

**Table 3:** Assessment methods for frailty, prevalence of frailty and outcomes in CLD patients reported in recent years.

Authors (Country and Year)	Patient Characteristics and Number	Diagnostic Method for Frailty	Proportion of Frailty (%)	Major Findings
Xu, et al. (USA, 2021) [84]	247 LC patients	LFI	26.7%	LFI may be more appropriate at capturing mortality risk than Karnofsky Performance Status.
Tsuchihashi, et al. (Japan, 2021) [60]	181 HCC patients (114 in the exercise group and 67 in the control)	LFI	Pre-frail or frailty; 79.8% (exercise group)and 71.6% (control)	In-hospital exercise improved frailty in HCC patients.
Nishikawa, et al. (Japan, 2020) [74]	341 CLD patients (LC, 122 cases (35.8%))	CHS criteria (Japanese version)	14%	Sarcopenia and frailty in CLD had common points and different points.
Saeki, et al. (Japan, 2020) [29]	291 patients (LC, 151 cases (51.9%))	CHS criteria (Japanese version)	27.8%	Frailty was an independent factor associated with osteosarcopenia.
Haugen, et al. (USA, 2020 [85])	882 LC patients (65 years or more, 16.6%)	LFI	65 years or more; 33.3% <65 years; 21.7%	Frailty was associated with nearly 2-fold increased risk of waitlist mortality, independent of age.
McKechnie, et al. (Canada, 2020 [67])	409 patients undergoing liver resection	modified FI	14.2%	High modified FI was an independent predictor for major postoperative complications.
Wang, et al. (USA, 2019) [80]	166 LC patients, 91 CLD patients9 1 non-CLD patients (control)	LFI	LC; 23%, CLD; 5%,non-CLD; 1%	The LFI involves external validity in non-LC patients.
Lai, et al. (USA, 2019) [76]	1044 LC patients who were listed or eligible for listing for liver transplantation	LFI	25%	Frailty is seen more frequently in LC patients with ascites or encephalopathy and independently associated with waitlist mortality.

LC, liver cirrhosis; HCC, hepatocellular carcinoma; CLD, chronic liver disease; LFI, liver frailty index; CHS, cardiovascular health study.

## Data Availability

Not applicable.

## Abbreviations

GS	grip strength
CLD	chronic liver disease
BCAA	branched-chain amino acid
LC	liver cirrhosis
JSH	Japanese Society of Hepatology
CT	computed tomography
BIA	bioelectrical impedance analysis
SMI	skeletal muscle index
AWGS	Asian Workng Group for Sarcopenia
WS	Walking speed
CC	calf circumference
EWGSOP	European Working Group for Sarcopenia in Older People
Zn	zinc
HCC	hepatocellular carcinoma
BMI	body mass index
LFI	Liver Frailty Index
LT	liver transplantation
